# Toward an Interdisciplinary Approach to Constructing Care Delivery Pathways From Electronic Health Care Databases to Support Integrated Care in Chronic Conditions: Systematic Review of Quantification and Visualization Methods

**DOI:** 10.2196/49996

**Published:** 2023-12-14

**Authors:** Luiza Siqueira do Prado, Samuel Allemann, Marie Viprey, Anne-Marie Schott, Dan Dediu, Alexandra Lelia Dima

**Affiliations:** 1 INSERM Unit U1290—Research on Healthcare Performance University Claude Bernard Lyon 1 Lyon France; 2 Pharmaceutical Care Research Group Department of Pharmaceutical Sciences University of Basel Basel Switzerland; 3 Pôle de Santé Publique Hospices Civils de Lyon Lyon France; 4 Catalan Institute for Research and Advanced Studies Barcelona Spain

**Keywords:** long-term care, electronic health care databases, patient pathway, data visualization, systematic review

## Abstract

**Background:**

Electronic health care databases are increasingly used for informing clinical decision-making. In long-term care, linking and accessing information on health care delivered by different providers could improve coordination and health outcomes. Several methods for quantifying and visualizing this information into data-driven care delivery pathways (CDPs) have been proposed. To be integrated effectively and sustainably into routine care, these methods need to meet a range of prerequisites covering 3 broad domains: clinical, technological, and behavioral. Although advances have been made, development to date lacks a comprehensive interdisciplinary approach. As the field expands, it would benefit from developing common standards of development and reporting that integrate clinical, technological, and behavioral aspects.

**Objective:**

We aimed to describe the content and development of long-term CDP quantification and visualization methods and to propose recommendations for future work.

**Methods:**

We conducted a systematic review following the PRISMA (Preferred Reporting Items for Systematic Reviews and Meta-Analyses) recommendations. We searched peer-reviewed publications in English and reported the CDP methods by using the following data in the included studies: long-term care data and extracted data on clinical information and aims, technological development and characteristics, and user behaviors. The data are summarized in tables and presented narratively.

**Results:**

Of the 2921 records identified, 14 studies were included, of which 13 (93%) were descriptive reports and 1 (7%) was a validation study. Clinical aims focused primarily on treatment decision-making (n=6, 43%) and care coordination (n=7, 50%). Technological development followed a similar process from scope definition to tool validation, with various levels of detail in reporting. User behaviors (n=3, 21%) referred to accessing CDPs, planning care, adjusting treatment, or supporting adherence.

**Conclusions:**

The use of electronic health care databases for quantifying and visualizing CDPs in long-term care is an emerging field. Detailed and standardized reporting of clinical and technological aspects is needed. Early consideration of how CDPs would be used, validated, and implemented in clinical practice would likely facilitate further development and adoption.

**Trial Registration:**

PROSPERO CRD42019140494; https://www.crd.york.ac.uk/prospero/display_record.php?RecordID=140494

**International Registered Report Identifier (IRRID):**

RR2-10.1136/bmjopen-2019-033573

## Introduction

### Background

Secondary use of patient data recorded during health care delivery in electronic health care databases (EHDs) has the potential to improve health care quality and reduce costs [1]. As long-term care consists of interactions with many health care providers for a long period [2,3], EHD data can be particularly useful for supporting decisions related to improving long-term care delivery [1]. With the increasing prevalence of chronic conditions worldwide, health care organizations can benefit from methods to link and transform EHD data from multiple sources into comprehensive descriptions of patients’ recent health status and health care use history [4-6]. These descriptions, which we refer to as data-driven *care delivery pathways* (CDPs), may apply numeric (quantification) or graphical (visualization) methods to synthesize information on the often-fragmented patients’ health care journeys [7]. The aim of CDPs is either to provide relevant clinical and contextual information to assist health care professionals (HCPs) and patients in making shared decisions on the course of treatment or to investigate sources of variation in health care use at the organization or system levels to inform quality improvement decisions. Providing feedback from routine care delivery via CDPs shows promise in reducing fragmentation and improving decision-making in chronic disease management [4]. CDPs are obtained from patients’ electronic records and retrospective evaluation of the CDPs with patients, in relation to their care goals and experiences, may help assess and work toward improving person-centered integrated care in long-term conditions [8].

Initial efforts to build such descriptions in different settings highlight the many challenges of developing CDPs that support clinical care in meaningful, reliable, and actionable ways. The challenges can be grouped into 3 domains: clinical, technological, and behavioral. First, the complexity of clinical situations may require information on multiple parameters relevant to a diverse range of decisions in the care process. CDPs require careful selection of key information depending on evidence-based clinical processes and treatment options, as well as data availability [9,10]. Second, developing the technology to access, link, clean, and produce comprehensible descriptions of these data and make them available at the point of care is a complex task. CDP visualization and quantification methods need to meet standards of data quality criteria of completeness, consistency, accuracy, reliability, and timeliness, among others [11]. Third, the aims of these methods can only be reached if their intended users act on this information in ways that optimize patients’ interactions with their HCPs and the decisions taken. Thus, CDP visualization and quantification methods, as with all complex interventions targeting human behaviors, should be designed to facilitate concrete actions by individuals (in this case, patients and HCPs) in specific contexts and moments in time [12]. Although these 3 domains have been partially considered in published work, no agreed approaches exist to deal with all the clinical, technical, and behavioral aspects of developing, evaluating, and implementing data-driven CDP visualization and quantification methods in long-term care. As health systems embark on similar projects that access data from EHD to guide the optimization of long-term care services, they would benefit from the learnings accumulated from the methods developed in recent years and how they considered these 3 domains. The insights gained could represent a basis for specifying minimal procedures to follow in project planning and conducting and reporting future projects and thus ensure more streamlined evidence synthesis in this field.

### Objective

The objective of this review was to describe and synthesize the different characteristics of quantification and visualization methods of data-driven long-term CDPs published in the scientific literature. We aimed to answer the following research questions:

1. What clinical information does the method use and how was it considered relevant?
2. What are the method’s development and implementation characteristics?
3. Which behaviors and interactions does the method aim to promote among users and how?

## Methods

### Overview

The protocol for this review was registered in PROSPERO (CRD42019140494) and published [7]. The review followed PRISMA (Preferred Reporting Items for Systematic Reviews and Meta-Analyses; see the checklist in Multimedia Appendix 1 [13]) guidelines [14] and included 7 steps: literature search, record screening and preliminary selection (title and abstract), full-text screening and final selection, data extraction, deductive-inductive analysis, critical appraisal, and data synthesis. In addition to the criteria mentioned in the protocol (involvement of stakeholders, source of funding, and conflicts of interest), critical appraisal was performed using the Quality Assessment Tool for Reviewing Studies With Diverse Designs (QATSDD) [15], a 16-item quality assessment tool designed to be applied to quantitative, qualitative, or mixed methods studies. The items received a score ranging from 0 (no information) to 3 (complete explanation). We attributed a “not applicable” (N/A) label when appropriate. We calculated the total score, ranging from 0 to 1, as the sum of the scores for applicable items divided by the number of applicable items. No other modifications were made to the initial protocol.

**Figure 1:**
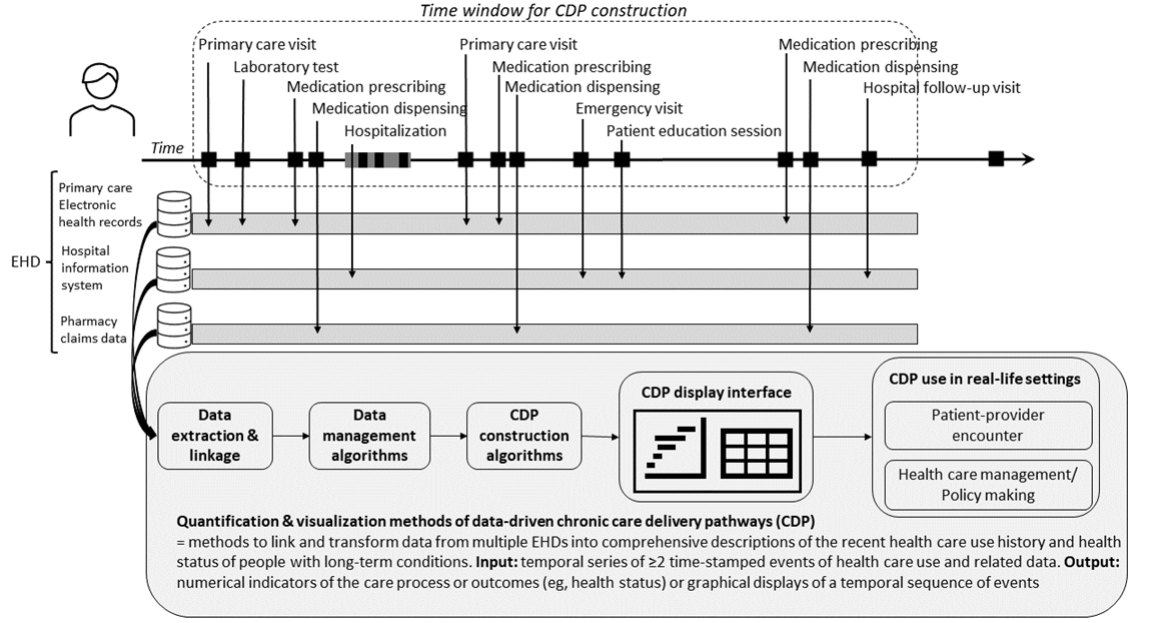
Definition of quantification and visualization methods of data-driven long-term care delivery pathways (CDPs) and illustration of a hypothetical example of a patient: In the time window selected for CDP construction, several health care use events occurred, including primary care visits, medication prescribing and dispensing, and hospitalization, and were recorded in 3 separate databases. For informing decisions in clinical encounters or at the organizational or policy level, this information needs to be extracted, linked across data sources, prepared for analysis, and results displayed in a user interface available in the intended situations. EHD: electronic health care database.

**Figure 2:**
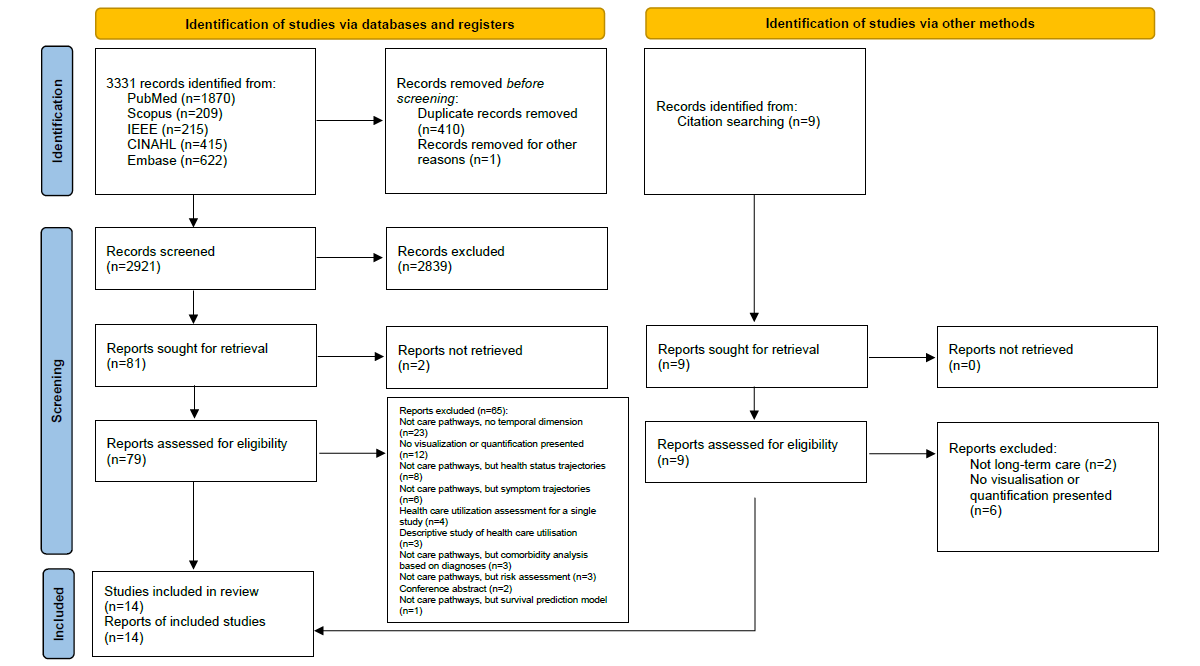
PRISMA (Preferred Reporting Items for Systematic Reviews and Meta-Analyses) flowchart describing the identification and screening of records and selection of included studies.

A literature search was performed using the PubMed (MEDLINE), Scopus, IEEE, CINAHL, and Embase databases. The terms searched were related to 3 topics: “data-driven” (Medical Subject Headings [MeSH] terms such as “electronic health record” and “data mining”); “clinical pathways” (MeSH terms such as “clinical pathway” and “disease management”); and “chronic conditions” (MeSH term “chronic diseases”). The search strategy is available in Multimedia Appendix 2. We considered peer-reviewed publications that (1) reported methods for visualization or quantification of data-driven chronic CDPs (including protocols and reports of study results; see the definition in Figure 1), (2) used data from people living with chronic conditions (ie, needing medical care for >12 mo), and (3) were published in English. No restrictions on publication date (up to March 2022), study design, population characteristics, type of health care facility, or level of care were applied. The following exclusion criteria were applied: studies that (1) aimed only to assess health care use over a specific period as part of a single research study, for example, as an outcome to evaluate health-related interventions, to describe populations or disease prevalence or as a proxy measure of disease aggravation risk; (2) did not mention population or data characteristics; (3) did not state that they analyzed data from people living with chronic conditions; (4) did not have full texts available; and (5) were not available in English. In addition, conference abstracts or abstract-only papers, systematic or narrative reviews, meta-analyses, and gray literature were not considered in the review.

Record screening was performed using the web-based systematic review management software Covidence (Veritas Health Innovation). After removal of duplicate studies, titles and abstracts were screened independently by 2 raters, as were full-text reports. Disagreements were discussed with a third rater until consensus was reached. We did not assess the interrater reliability for screening. We checked the reference lists for additional relevant studies.

### Data Extraction

An electronic data extraction form was used to extract information from included reports on study characteristics (authors, title, type of study, year and country of study, objective, and research questions) and population characteristics (number of patients, age, gender, chronic conditions). Moreover, for the clinical domain, we extracted information on clinical and cost outcomes and the clinical information presented on the proposed interfaces, if present, or data summaries, along with the description of how authors evaluated the relevance of the information (eg, consulting with experts). We also extracted data on method development and validation and stated users and use case scenarios. Data extraction was performed by a single rater and reviewed by a second rater, whereas quality assessment was performed by 2 raters, independently.

In the technological domain, we performed the deductive-inductive content analysis to appraise method development and validation. Inductive analysis includes open coding and creating categories directly from the analyzed text, whereas deductive analysis uses existing data applied to a new context [16]. To perform the deductive analysis, we used the framework proposed by Moreno-Conde et al [17] to describe projects defining clinical information models (CIMs). CIMs are technical specifications that define how information is organized and described within electronic health record (EHR) systems, thus facilitating data entry, storage, exchange, analysis, and display. CIMs are developed based on standard reference models and clinical terminologies and work toward a locally implementable structure and semantics that are consistent with these standards, thus enabling interoperability. The description of technological development included 7 steps: scope definition, domain analysis, tool design, definition of tool specifications, validation, publishing and maintenance, and governance [17]. We extracted descriptions of the CDP methods corresponding to these steps. If these descriptions included information that could not be mapped onto these 7 categories, we constructed new categories inductively. The resulting updated framework (described in the Results section) was discussed among 2 coders until consensus was reached.

In the behavioral domain, considering the use of health information systems such as hospital information systems (HISs) and other clinical software as health-related processes, we applied the Action, Actor, Context, Target, Time (AACTT) framework to analyze the use scenarios presented and describe user behaviors. AACTT is a behavior specification framework applicable to implementation interventions in health care to clarify the behaviors of stakeholders across multiple levels of the health system. An action is the behavior that needs to change or occur, in terms that can be observed or measured; an actor is the person (or persons) that does or could do the actions targeted; a context is the physical location, emotional context, or social setting in which the action is performed; a target is the person (or persons) with or for whom the action is performed; and time specifies when the action is performed (time, date, or frequency) [12].

## Results

### Overview

We identified 3331 records across the databases searched, resulting in 2821 records after duplicate removal and 81 records after title and abstract screening. Citation searching led to the identification of 9 records that reached full-text screening. Finally, 14 studies were included from 14 reports (Figure 2 [13]).

Of the 14 studies, 10 (71%) were performed in English-speaking countries: the United States [6,18-21], United Kingdom [22-24], Australia [25], and New Zealand [26]; the rest (n=4, 29%) were performed in Italy [27], China [28], Finland [29], and Germany [30]. A total of 11 (79%) articles were published after 2011 [6,18-24,28-30], and 3 (21%) articles were published by the same group [6,18,21]. We identified 1 (7%) protocol [24], 12 (86%) descriptive studies [6,18-29], and 1 (7%) validation study [30]. All but 1 (7%) study had descriptive objectives, that is, presented the method and its development. Although all studies focused on support systems for long-term care, the objectives were described using different terms from different perspectives: clinical decision support system [29], decision support system focused on care planning [25], data aggregation from different sources in the continuum of care [30], care coordination (care flow management) system [27], linkage system between different data sets [23], system to predict health status transitions [20], framework and ontology for chronic disease management [19,26], and systems to build and visualize clinical pathways [6,18,21,22,24,28]. A total of 8 (57%) studies and the protocol reported a method validation process [19,20,22-24,26,28,29]. In 1 (7%) study, evaluation with patients and clinicians in real-life clinical settings was reported [30]. The studies targeted different chronic conditions: type 1 and type 2 diabetes [27], type 2 diabetes and hypertension [24], ankylosing spondylitis [23], glioblastoma multiforme [19], prostate cancer [22], traumatic brain injury [29], chronic kidney disease [18], rheumatoid arthritis [30], and hypertension [20,26]; 4 (29%) studies targeted patients with multiple chronic conditions [6,21,25,28]. All data collected from the studies are available in Multimedia Appendix 3 [5,6,19-30]. The characteristics of the included studies are detailed in Table 1.

**Table 1:** Characteristics of selected studies.

Study, year	Title	Country	Type of study	Objectives	Studied population	
					Patients, n	Conditions
Warren et al [25] 1999	Chronic disease coordinated care planning: flexible, task-centered decision support	Australia	Descriptive	Descriptive: CPOL^a^, a decision support system for chronic care planning at SA HealthPlus by integrating relevant information flows at the point-of-care user interface and architecture	4000	High-use patients in South Australia in 10 groups including diabetes, cardiac, aged care, and lung disease
Panzarasa et al [27] 2004	A careflow management system for chronic patients	Italy	Descriptive	Descriptive: infrastructure (CfMS^b^) for enabling the cross-organizational communication process of chronic disease management in diabetes care	Not reported	Type 1 and type 2 diabetes
Mabotuwana and Warren [26] 2010	ChronoMedIt—a computational quality audit framework for better management of patients with chronic conditions	New Zealand	Descriptive and validation	Descriptive: ChronoMedIt^c^ is a framework that takes temporal considerations into account when formulating and executing audit criteria in chronic disease management Validation: to apply the framework to 2 practices’ data sets to detect patients with suboptimal management	1286	Hypertension
Husain et al [23] 2012	HERALD (Health Economics using Routine Anonymised Linked Data)	United Kingdom	Descriptive	Descriptive: procedures linking patient-derived questionnaire data with routinely collected information and secondary care clinical data sets to conduct health economics analyses Validation: to map patients journeys in an ankylosing spondylitis cohort in 3 different settings (general practitioner, outpatients, and inpatients)	715	Ankylosing spondylitis
Hsu et al [19] 2012	Context-based electronic health record: towards patient specific healthcare	United States	Descriptive	Descriptive: AdaptEHR, a context-based EHR^d^ using biomedical ontologies and (graphical) disease models as sources of domain knowledge to identify relevant parts of the free-text record to extract, aggregate, map on ontologies and display in the patient record for different users depending on their information needs to inform medical decision-making Validation: to implement the framework in a system called AdaptEHR to present and synthesize information from neuro-oncology patients	283	Glioblastoma multiforme (brain cancer)
Sun et al [20] 2013	Predicting changes in hypertension control using electronic health records from a chronic disease management program	United States	Descriptive and validation	Descriptive: approach for predicting the risk and timing of transitions (deterioration or improvement) in hypertension control using all available clinical information from electronic health records (demographics, diagnoses, medications, and laboratory results) and physician judgment of hypertension control status, using a feature selection strategy to identify relevant predictors Validation: to evaluate the prediction approach on a patient cohort in a chronic disease management program, the Vanderbilt MHT^e^	1294	Hypertension
Bettencourt-Silva et al [22] 2015	Building data-driven pathways from routinely collected hospital data: a case study on prostate cancer	United Kingdom	Descriptive and validation	Descriptive: to propose a framework for building and visualizing individual data-driven patient-centric pathways from routinely collected hospital data for prostate cancer Validation: to evaluate the completeness and utility of the generated pathways for investigating biomarker trends	1904	Prostate cancer
Zhang and Padman [5] 2015	On clinical pathway discovery from electronic health record data	United States	Descriptive	Descriptive: iterative, practice-based clinical pathway development process that integrates health IT and domain knowledge and includes elicitation of practice patterns (candidate clinical pathways) from electronic health records data about the sequence of patients’ visits to the clinic represented by a 1-dimensional Markov chain	1624	CKD^f^
Zhang and Padman [6] 2016	Data-driven clinical and cost pathways for chronic care delivery	United States	Descriptive	Descriptive: approach to incorporate medical costs in the clinical pathways of patients with multiple chronic conditions Validation: to compare a cost-centered perspective and a clinically focused perspective to show similarities and differences in the categorization of pathways and patient subgroups	288	CKD stage 3, diabetes, and hypertension
Zhang and Padman [21] 2017	An interactive platform to visualize data-driven clinical pathways for the management of multiple chronic conditions	United States	Descriptive	Descriptive: prototype of an interactive visualization platform on treatment of patients with multiple chronic conditions (clinical pathways); design, development, and implementation	1084	CKD, hypertension, and diabetes
Litchfield et al [24] 2017	Can process mining automatically describe care pathways of patients with long-term conditions in UK primary care? A study protocol	United Kingdom	Protocol	Descriptive: algorithms for automated process mining for senior practice staff and commissioning groups to understand care delivery processes (method and development) Validation: to compare the results of automated process mining with traditional process mapping methods in patients with hypertension or type 2 diabetes at 4 primary care practices	4000 (estimated)	Type 2 diabetes and hypertension
Guo et al [28] 2019	Visual progression analysis of event sequence data	China	Descriptive and validation	Descriptive: ET2^g^ is a visual progression analysis technique and system, including a stage analysis algorithm and a system for visual query and interrogation Validation: to evaluate the effectiveness of ET2 in identifying evolution through stages with real-world data compared with known ground truth; collect expert feedback on whether the output is meaningful, informative, easy to use, interpretable, and readable	145	Cardiovascular disease
Umer et al [29] 2019	A decision support system for diagnostics and treatment planning in traumatic brain injury	Finland	Descriptive and validation	Descriptive: decision support system for diagnostics and treatment planning in traumatic brain injury. Modules and their functionalities, architecture, and development (requirement elicitation, implementation) Validation: to evaluate the usability of the decision support systems in 2 clinical settings	400 (training data)+60 (validation study)	Traumatic brain injury
Richter et al [30] 2021	The PICASO^h^ cloud platform for improved holistic care in rheumatoid arthritis treatment—experiences of patients and clinicians	Germany	Validation	Validation: evaluate an information and communication platform using an evaluation framework, in a 6-month proof-of-concept study in clinical routine care of patients with rheumatoid arthritis and their providers	30	Rheumatoid arthritis

^a^CPOL: Care Planning On-Line.

^b^CfMS: Careflow Management System.

^c^ChronoMedIt: Chronological Medical audit.

^d^EHR: electronic health record.

^e^MHT: MyHealthTeam.

^f^CKD: chronic kidney disease.

^g^ET2: EventThread 2.

^h^PICASO: Personalised Integrated Care Approach for Service Organisations and Care Models for Patients with Multi-Morbidity and Chronic Conditions.

### Critical Appraisal

Of the 14 studies, 8 (57%) studies involved stakeholders in the development [19,22,24-26,28-30]. Although 2 (14%) studies declared to have received public and private funding [20,25], 4 (29%) did not declare funding [18,21,22,24], 7 (50%) declared to have received public funding [19,23,26-30], and 1 (7%) declared to have received no funding [6]. A total of 6 (43%) studies included a conflicts of interest section and declared no interests [6,20,22-24,30], and 8 (57%) studies did not specify [5,19,21,25-29]. Four QATSDD items were applicable to all or most of the studies: “Statement of aims/objectives in main body of report,” “Clear description of research setting,” “Evidence of user involvement in design,” and “Strengths and limitations critically discussed.” The QATSDD appraisal results are available in Multimedia Appendix 4 [5,6,19-30]. The scores ranged between 0.33 and 1 (mean 0.64, SD 0.16).

### Clinical Domain: What Information Was Used and How Was It Considered Relevant?

The most common clinical aim was to provide visualizations of longitudinal health care use data to optimize clinical pathways (Table S1 in Multimedia Appendix 5) [6,18,22,23]; other aims were care planning [25], detecting patients with chronic conditions on suboptimal management [26], or informing care decision-making [19]. Half of the studies (7/14, 50%) [19,22,24,25,27,29,30] used data from multiple EHDs, including EHRs and HISs. The most common relevance criteria for data selection were consultations with experts [19,20,22,24-26,28,29] and guidelines for the targeted chronic condition [25,27]. One study [30] reported an evaluation in clinical practice with patients and clinicians, and the evaluated outcomes were both clinical (patient-reported outcome measures, eg, functional ability and disease activity) and related to user experience (acceptability, usability, user satisfaction, and clinical relevance of the platform). Some records proposed real-life evaluation criteria for future work, ranging from comparing HCPs’ performance with and without the proposed system [19,25,29], comparing care maps produced by the system to those produced using traditional process mapping methods [24], to using qualitative methods such as think-aloud and focus groups [21]. Although 1 (7%) record reported that the system provided feedback on cost [25] and 1 (7%) built care pathways using EHR and medication cost data [6], none reported cost outcomes for evaluation.

### Technological Domain: How Were the Methods Developed and Implemented?

Of the 7 categories by Moreno-Conde et al [17], 6 were identified; the exception was *publishing and maintenance*, which was not reported on by any study. Two new categories were identified through inductive content analysis: *dataflow and transformation* (explicitly describing how different data sets were linked or the linkage algorithms that were used to trace the final data items, to understand how these tools could be integrated into existing EHRs, and to consider interoperability) and *data protection* (describing measures taken to protect patients’ data, such as anonymizing or pseudoanonymizing patient data to ensure protection of private or sensitive data)*.* These categories included up to 3 subcategories each, resulting in 14 technological characteristics; the 14 studies reported information referring to a median of 7 (IQR 5.5) subcategories, ranging from 3 to 11 (Table 2).

**Table 2:** Technological characteristics of the care delivery pathway quantification and visualization methods selected (n=14).

Categories (n=14)		Warren et al [25] 1999	Panzarasa et al [27] 2004	Mabotuwana, and Warren [26] 2010	Husain et al [23] 2012	Hsu et al [19]	Sun et al [20] 2013	Bettencourt-Silva et al [22] 2015	Zhang and Padman [5] 2015	Zhang and Padman [6] 2016	Zhang and Padman [21] 2017	Litchfield et al [24] 2017	Guo et al [28] 2019	Umer et al [29] 2019	Richter et al [30] 2021	Studies reporting, n (%)	
**Scope definition leading to selection of the domain and selecting relevant experts**																	
	Information on the domain to be covered and whether the scope is local or wider are presented	✓	✓	✓	✓	✓	✓	✓	✓	✓	✓	✓	✓	✓	✓	14 (100)	
	The study involved a group of experts based on the care setting, health care activities, and clinical requirements	✓		✓		✓		✓		✓	✓	✓	✓	✓	✓	10 (71)	
	Expected uses or use case scenarios are presented		✓	✓							✓		✓	✓		5 (36)	
**Analysis of the information covered in the specific domain**																	
	Clinical scenarios, workflows, and users are understood to determine the data items to be used in the method		✓			✓		✓						✓		4 (29)	
	Existing systems are described (how they have been implemented and documented)		✓					✓					✓	✓		4 (29)	
Design of the tool: the set of attributes associated with the method is detailed		✓	✓	✓		✓	✓	✓			✓	✓	✓	✓	✓	11 (79)	
Implementable technical specification is described			✓	✓		✓		✓			✓		✓	✓	✓	8 (57)	
**Validation**																	
	The study presents prototype screens	✓		✓		✓	✓	✓	✓	✓	✓		✓	✓	✓	11 (79)	
	The method is validated (eg,: training or testing data, pilot study, implementation test, etc)			✓			✓	✓				✓	✓	✓	✓	7 (50)	
**Governance**																	
	There is an organization responsible for developing and maintaining the method														✓	1 (7)	
	If applicable, this organization oversees quality review, publication, and relationships with other projects working on the same domain														✓	1 (7)	
**Dataflow and transformation**																	
	Linkage between data sets is described	✓	✓	✓		✓	✓	✓	✓		✓	✓			✓	7 (7)	
	The architecture of the tool is presented	✓	✓	✓		✓	✓	✓	✓		✓	✓		✓	✓	8 (57)	
Data protection for the development is described				✓	✓		✓	✓	✓	✓		✓	✓		✓	9 (64)	
Subcategories with information reported, n (%)		6 (43)	7 (50)	10 (71)	3 (21)	7 (50)	6 (43)	11 (79)	3 (21)	4 (29)	6 (43)	5 (36)	10 (71)	10 (71)	11 (79)	N/A^a^	

^a^N/A: not applicable.

All studies (N=14) included information on the domain to be covered and whether the scope of the presented system was geographically local or wider. Although 10 (71%) studies mentioned involving a group of experts or discussing with clinicians during the method development [6,19,21,22,24-26,28-30], 2 (14%) studies [24,29] provided details about how expert feedback would be obtained and applied. In 1 (7%) study, interviews with 11 specialists were conducted to develop the first version, and later an iterative process of feedback and development was undertaken with 5 specialists [29]. The study protocol stated that development would be iterative, with a clinical expert and the informatics lead [24]. Clinical scenarios and workflows were presented in 4 (29%) studies [19,22,27,29]. The characteristics of the existing systems, such as how they were implemented and documented, were presented in 4 (29%) studies [22,27-29]. Most studies (11/14, 79%) detailed the set of attributes associated with the method [19-22,24-30]. Implementable technical specifications were presented in 8 (57%) studies [19,21,22,26-30]. Validation or testing was performed using different strategies: applying the developed tool to a cohort of patients and evaluating key performance indicators (4/14, 29%) [20,22,23,26], comparing between using the tool and not using the tool (2/14, 14%) [24,29], adding the system to an existing EHR system and collecting expert feedback through an initial usability test (1/14, 7%) [19], or through showing the tool to experts and performing qualitative interviews (n=1, 7%) [28]. In 1 (7%) study, a real-life evaluation was performed [30]. Another (n=1, 7%) study reported on the evaluation of a platform held by a consortium responsible for developing and maintaining the method [30]. A total of 6 (43%) studies explicitly described how different data sets were linked or the linkage algorithms [19,22,23,26,28]. Although the architecture or a conceptual model of the system was provided in 5 (36%) studies [20,22,25,26,29], system architecture and data models, expressed in Unified Modeling Language, XML, or as ontologies, were reported in 6 (43%) studies [22,26-29]. In addition, 1 (7%) study described and discussed data quality in EHR [22], and 1 (7%) study mentioned using the Health Level 7 and Fast Healthcare Interoperability Resources standards to enable data exchange with other systems [30]. In 8 (57%) studies, anonymized [6,18,20-23,26] or pseudoanonymized patient data were used [24]. In 1 (7%) study, a publicly available anonymized data set was used [28], and in 1 (7%) study, the platform was cloud based following European standards to ensure data security [30].

### Behavioral Domain: Actions and Interactions to Improve Care Delivery

In total, 3 (21%) studies presented use scenarios with sufficient detail to identify the actor (who would use the method), activities, contexts, moments, and target (Table 3).

**Table 3:** Behavioral elements present in the care delivery pathway quantification and visualization methods selected. The text describing the scenarios was extracted from the original articles.

Reference and extracted scenario description			Actor	Action	Context	Time	Target
**Panzarasa et al [27]** **2004**							
	**Scenario 1**						
		“An important task performed by the Cf is the automatic evaluation of home monitoring data, as soon as they are sent to the diabetes management center, in order to detect potential critical situations that should be notified to the care providers or that could require further clinical investigations. This analysis calculates several descriptive statistics (i.e., arithmetic means, standard deviations and the highest and lowest values in a given period of time), performs data interpretation through the extraction of patterns of clinical importance and consistency checking. If the Cf notices that the patient is not responding in the expected way to the therapy (e.g., hyperglycemia and hypoglycemia are too frequent) it generates a guideline-based suggestion to the physician about the need of a therapy modification.”	Physician	To modify treatment	Patient’s home	After system alert	Patient
	**Scenario 2**						
		“In order to help physicians and patients in the management of the long-term screening, the CfMS schedules periodic visits based on the patient’s care process history”	Patient	To schedule medical visit	Patient’s home	Periodically	Patient
	**Mabotuwana and Warren [26]** **2010**						
	**Scenario 1**						
		“Awareness of immediate cases – identification of those patients that, at a particular moment in time, are out of supply of an indicated medication. In the first instance, the action is to treat the non-adherence as inadvertent and recall the patient and/or simply prescribe as indicated at the next opportunity. This includes not just patients with lapsed medications, but also those whose circumstances have changed (e.g., due to development of a co-morbidity) and thus require additions to previous therapy”	GP^a^	Treats nonadherence or add new treatments	Consultation	When identified	Patient
	**Scenario 2**						
		“Opportunity for communication with those with poor supply profiles – at some point it becomes logical to look to a lack of concordance between doctor and patient, and/or to the ability of the patient to achieve adherence for other reasons. Low Medication Possession Ratio over an extended time period and repeated lapses in medication supply indicate the need for improved communication between GP and patient; possibly the clinician needs to engage the patient more in a joint ‘‘problem-solving” approach in relation to underlying adherence barriers.”	GP	To engage in “problem-solving” approach	Consultation	Not clear	Patient
	**Scenario 3**						
		“Opportunity to critique GPs on their adherence to established guidelines and compare practices on specified criteria – for example, the JNC7 hypertension guideline recommends ACEi/ARB medication as compellingly indicated therapy for patients with comorbid hypertension and chronic kidney disease. If an agreed set of audit criteria can be established, this form of reporting also provides an opportunity to compare GP practices (as we have done here with two practices) in an attempt to provide feedback to the GPs to improve the management of their patients with chronic conditions.”	Not identified	To give feedback to GPs on guideline adherence	Not identified	Not identified	Patient
**Warren et al [25]** **1999**							
	**Scenario 1**						
		“Users are actively alerted to review relevant guidelines through the several mechanisms: 1) Flags on observations. Right-clicking these observations or the flags will invoke the relevant guideline. Relevant guidelines will appear where observations are recorded (such as in the Initial Medical Assessment form) and in the “heads up” patient summary. 2) Flags on services. 3) Explicit save-time warnings. In some specific cases like vaccination the user will be prompted to consider a particular guideline before exiting the client application.”	User (GPs)	To review guidelines (not specified)	Software use	When flags and warnings are shown	Patient

^a^GP: general practitioner.

Other studies mentioned intended uses in general terms (eg, “improve shared decision-making” or “help clinicians in making decisions”). Although 3 (21%) articles did not specify who the intended end users were [20,23,24], 6 (43%) stated that the end users were physicians (primary care providers or specialists) [19,22,25,26,28,29] and 5 (36%) included patients and families as end users, in addition to physicians and managers [6,18,21,27,30].

Panzarasa et al [27] mentioned 2 scenarios. The first described a physician using a warning generated by a care flow management system from home monitoring of blood glucose and intervening to modify treatment with the patient, if needed. The second described patients and physicians using suggestions for periodic medical visits issued by the system based on integrated guidelines and health care use data to plan their care. Mabotuwana and Warren [26] described 3 scenarios. In the first, the system detects patients who are out of medication supply and warns the physician to either address nonadherence or provide a prescription in the next consultation. The second described the system as allowing physicians to become aware of patients with whom they might have to improve communication and engage in a “problem-solving” approach, also by detecting patients with low medication availability. In the third scenario, they described the system as providing audit criteria and relevant data to allow for the assessment of physicians’ adherence to guidelines and compare practices. Warren et al [25] described a scenario in which the system alerts users (physicians) to review guidelines.

## Discussion

### Principal Findings

This review takes stock of pioneering work on methods to quantify and visualize CDPs and describes the characteristics of the resulting tools and their development from 3 key perspectives: clinical, technological, and behavioral. We identified 14 studies targeting different chronic conditions and clinical settings in 8 countries, indicating the international reach of this emerging area. From a clinical perspective, the main aim and expected benefit of these methods were to improve physicians’ decision-making by enhancing the interpretation of individual-level data; to this end, clinical guidelines and collaborating clinicians provided the relevant input for method development. From a technological perspective, most studies presented details on technological development, system architecture, and data sources. Few studies provided information on the validation processes, and only 1 real-life evaluation was performed. Most papers reported stakeholder involvement before or during development. From a behavioral perspective, 3 of the 14 studies mentioned possible actions intended to follow from accessing CDP data or visualizations, referring to adjusting treatment, care planning, supporting treatment adherence, or clinicians’ guideline adherence. All but one of the studies were descriptive reports of early development work and described similar development steps. Nevertheless, the substantial variation in the types of information reported suggests a need for structuring common methodological standards for guiding future projects and facilitating evidence synthesis.

From a clinical perspective, the reviewed studies presented views or prototype screens, but clinical aims and use scenarios were mostly insufficiently described to ascertain their applicability to clinical contexts. This represented a challenge for the review process, which highlights the value of comprehensive and standardized descriptions for future evidence synthesis. Most studies focused on condition-specific biomarkers and health care use history to build the visualization to enhance specialists’ clinical decisions, and the concept of team-based long-term care and features that could improve provider-provider or patient-provider communication were not common. Only 2 studies mentioned cross-organizational communication to improve the management of a chronic condition [27,30], which is essential to integrated care. The studies reported systems that were not evaluated in real-life settings to assess the impact of their implementation on care organization, quality, or effectiveness. Our search for subsequent articles that described further evaluation of the analyzed methods did not identify additional records.

From a technological perspective, although clinical information is readily available in current EHR systems, the diversity in patient data and the lack of interoperability between different health care organizations’ HISs are still challenges to the secondary use of patient data [1,5]. As observed in this review, the main data source for most studies was EHRs. When other HISs were used, data acquisition processes and linkage methods were not always presented. Other system characteristics such as architecture and data security measures were available in some reports, such as in the work of Bettencourt-Silva et al [22]; they reported a methodology to build data-driven pathways using patient-centric data from different databases and presented the data extraction process, the methodology, the building of an operational data store, the pathway and the analysis engine, and the visualization software. Finally, this was the only study in our sample to report and discuss quality indicators. The absence of crucial details concerning data acquisition, interdatabase linkage, data quality, and security not only posed a challenge in summarizing the methods outlined in this review but also hindered their reproducibility.

From a behavioral perspective, the technological innovations analyzed here did not clearly specify actions or use scenarios and did not always identify end users. This is common in health services research and explains in part the reason that suboptimal clinical practices persist and are associated with avoidable morbidity and mortality despite the potential benefits of uptake of innovations to care organization and practice [12]. It also posed a barrier to the review process, making it harder to fully understand the context in which these methods were meant to be applied. Furthermore, specifying behaviors represents a starting point for identifying their barriers and facilitators (at individual, team, and system levels) and strategies or techniques to promote behavior change in the agreed direction, for example, by features integrated into the tool or by additional user training. Using longitudinal health care visualizations and decision support systems, care coordination can be promoted at the HCP level through functions such as messaging systems to provide cues for action or feedback on behavior from encounter history [31]. The studies included did not mention strategies to promote intended behaviors or user training, which suggests that integration into clinical care was not envisaged yet. Moreover, in the studies included, patients were not systematically considered as possible users, even when aims included improving the shared decision-making process at the point of care. Considering that access to actionable information can lead to better chronic disease management [32,33], including patients as end users and integrating their preferences in the development phase of such tools have the potential to improve the shared decision-making process.

**Figure 3:**
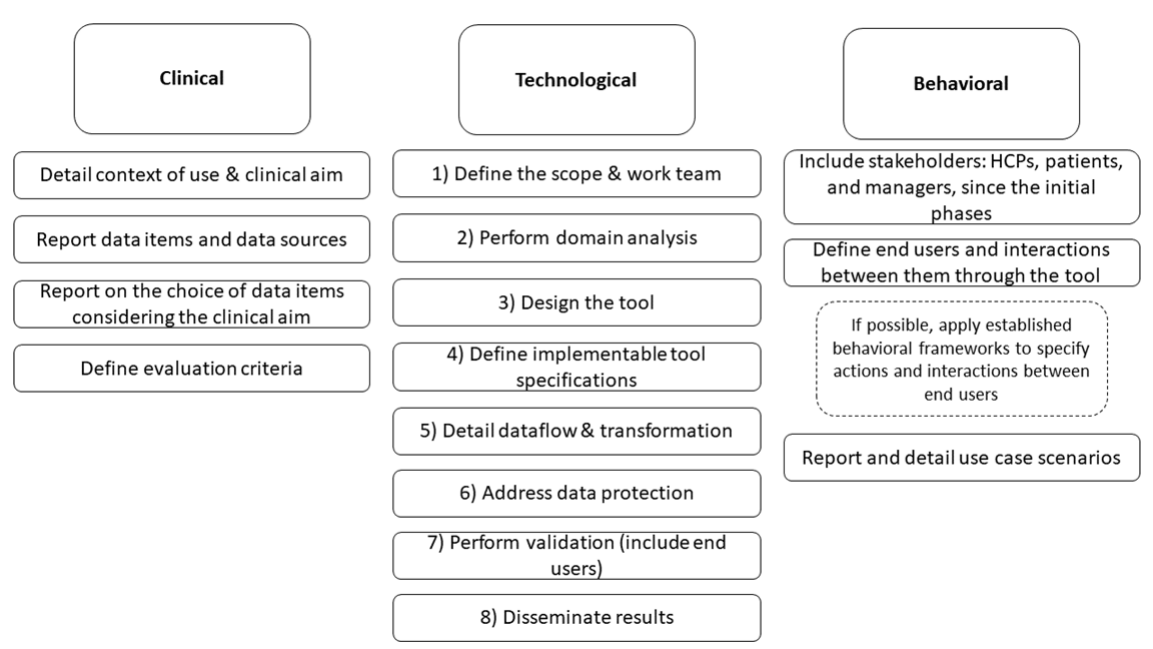
Key points to developing new data-driven care delivery pathway visualization and quantification methods.

The differences among the included studies in terms of the type of information reported highlight the need for common reporting guidelines and the development and adoption of best practices in this research area. Following the adapted technology development framework we applied in this review [17], we propose that new projects developing data-driven long-term CDP visualization and quantification methods could benefit from descriptions including clinical (aim, information used, relevance, and evaluation criteria); technological (which could be followed sequentially in the method development); and behavioral aspects (identifying end users and their actions expected following the established behavioral frameworks, such as the AACTT; Figure 3). Standardizing terminology for describing the different aspects is paramount for facilitating evidence synthesis and the evolution of this field. The development of these methods could follow a similar process with CIM development, including (1) defining scope and the work team comprising potential end users (HCPs, patients, and managers); (2) performing a domain analysis; (3) designing the tool; (4) defining implementable tool specifications; (5) detailing dataflow and transformation; (6) addressing data protection; (7) performing validation by end users; and (8) disseminating results. Moreover, we consider that the development of data-driven long-term CDP frameworks could benefit from following established frameworks for developing complex health interventions [34-36], which would improve the design and reporting of such system-level innovations.

### Limitations

Our review presents several limitations that would need to be addressed in future work. First, although we used several keywords and terms in different databases, it is likely that our search might have missed relevant publications due to the diversity of terminologies used in describing CDPs. This highlights the importance of developing common terms and definitions for this type of work. Second, because health care technology is a rapidly evolving field, not all technologies developed are disseminated in scientific publications. Our review did not include studies that were not published as peer-reviewed papers because it focused on projects that aimed to produce and disseminate scientific evidence to support their tool. Therefore, our results should be interpreted as a synthesis of the best available evidence. Future work could review other types of literature to identify a broader range of methods developed in more diverse contexts. Third, we focused on CDP description methods applied to people living with chronic conditions, in view of the importance of care coordination in long-term care. Thus, we have excluded visualization methods applied in other settings [37-40], which may have the potential to be applied in long-term care. Future applications of these methods may consider the specific requirements identified in this review relevant to these use scenarios.

### Conclusions

In conclusion, this review is the first to describe the emerging field of data-driven long-term CDP visualization and quantification methods, systematically examine published research, and propose ways to structure the conduct and reporting of such studies in the future. These methods share common elements with health information technology–supported clinical pathways, but they represent a distinct category of innovations given the use of retrospective EHD data and the data display that considers the sequence of events in a timeline (temporal dimension). Moreover, we believe that data-driven long-term CDP visualization tools can be used to enable integrated care, combining different data elements in comprehensive views to be used at the point of care. To address the issue of different terminologies used to describe CDPs, we propose data-driven care delivery pathway as a unifying term and welcome further clarifications and agreement on the terminology. This review has allowed for the description of a research area that is under development and subject to multiple challenges, which require concerted efforts for standardization.

## Appendix

Multimedia Appendix 1 PRISMA (Preferred Reporting Items for Systematic Reviews and Meta-Analyses) checklist.

Multimedia Appendix 2 Search strategy.

Multimedia Appendix 3 Critical appraisal.

Multimedia Appendix 4 Clinical aspects of the care delivery pathway quantification and visualization methods selected.

Multimedia Appendix 5 Codebook and data extraction table.

## Abbreviations

AACTT: Action, Actor, Context, Target, Time
CDP: care delivery pathway
CIM: clinical information model
EHD: electronic health care database
EHR: electronic health record
HCP: health care professional
HIS: hospital information system
MeSH: Medical Subject Headings
PRISMA: Preferred Reporting Items for Systematic Reviews and Meta-Analyses
QATSDD: Quality Assessment Tool for Reviewing Studies With Diverse Designs
