# Supplementary material for: Toward an Interdisciplinary Approach to Constructing Care Delivery Pathways From Electronic Health Care Databases to Support Integrated Care in Chronic Conditions: Systematic Review of Quantification and Visualization Methods
Source: J Med Internet Res. 2023 Dec 14;25:e49996. doi: 10.2196/49996 (PMC10755664; doi:10.2196/49996)
Supplement: Multimedia Appendix 2 [file jmir_v25i1e49996_app2.docx]

COMPLETE SEARCH STRATEGY

| Category | Medical Subject headings (MeSH) | CINAHL Search | EMBASE | Keywords |
| --- | --- | --- | --- | --- |
| Data-driven | Electronic health record; data mining; machine learning; clinical decision support systems; analysis, cluster; medical informatics application | MH Electronic Health Records OR MH Data Mining OR MH Nursing Informatics OR MH Machine Learning OR MH Decision Support Systems, Clinical OR MH Cluster Analysis OR MH Medical Informatics OR MH Computer Graphics OR MH Algorithms OR TI Data-driven OR TI visualisation OR TI computer graphics OR TI process mining OR TI data mining OR TI visualization OR TI supervised learning OR TI unsupervised learning OR TI practice based OR TI modelling OR TI mapping OR TI cluster* OR TI data analys* OR AB Data-driven OR AB visualisation OR AB computer graphics OR AB process mining OR AB data mining OR AB visualization OR AB supervised learning OR AB unsupervised learning OR AB practice based OR AB modelling OR AB mapping OR AB cluster* OR AB data analys* | (Electronic health record or data mining or machine learning or clinical decision support systems or analysis, cluster or medical informatics application).sh. or (Data-driven or visualisation or computer graphics or process mining or data mining or visualization or supervised learning or unsupervised learning or practice based or modelling or mapping or cluster* or data analys*).ti. or (Data-driven or visualisation or computer graphics or process mining or data mining or visualization or supervised learning or unsupervised learning or practice based or modelling or mapping or cluster* or data analys*).ab. | Data-driven OR visualisation OR computer graphics OR process mining OR data mining OR visualization OR supervised learning OR unsupervised learning OR practice based OR modelling OR mapping OR cluster* OR data analys* |
| Clinical pathways | Clinical pathways; delivery of health care, integrated; clinical practice pattern; disease management; care management, patient | MH Critical Path OR MH Health Care Delivery, Integrated OR MH Practice Patterns OR MH Disease Management OR MH Patient Care Plans OR TI Clinical course OR TI integrated care OR TI care map OR TI care pathway OR TI care plan OR TI treatment plan OR TI patient journey OR TI patient flow OR TI clinical redesign OR TI integrated care OR AB Clinical course OR AB integrated care OR AB care map OR AB care pathway OR AB care plan OR AB treatment plan OR AB patient journey OR AB patient flow OR AB clinical redesign OR AB integrated care | (Clinical pathways or delivery of health care, integrated or clinical practice pattern or disease management or care management, patient).sh. or (Clinical course or integrated care or care map or care pathway or care plan or treatment plan or patient journey or patient flow or clinical redesign or integrated care).ti. or (Clinical course or integrated care or care map or care pathway or care plan or treatment plan or patient journey or patient flow or clinical redesign or integrated care).ab. | Clinical course OR integrated care OR care map OR care pathway OR care plan OR treatment plan OR patient journey OR patient flow OR clinical redesign OR integrated care |
| Chronic conditions | Chronic diseases; chronic illness | MH Chronic Disease OR TI Integrated chronic care OR AB Integrated chronic care |  | Integrated chronic care |

EQUATIONS

**MEDLINE**

((data-driven[Title/Abstract] OR health information[Title/Abstract] OR data analys*[Title/Abstract] OR computer graphics[MeSH Terms] OR visualization[Title/Abstract] OR machine learning[MeSH Terms] OR data mining[MeSH Terms] OR clinical decision support systems[MeSH Terms] OR medical informatics application[MeSH Terms] OR algorithm[MeSH Terms] OR supervised learning[Title/Abstract] OR unsupervised learning[Title/Abstract] OR analysis, cluster[MeSH Terms] OR practice-based[Title/Abstract] OR electronic health record[MeSH Terms] OR clinical decision support systems[Title/Abstract] OR process mining[Title/Abstract] OR data mining [Title/Abstract] OR machine learning [Title/Abstract] OR medical informatics application[Title/Abstract] OR cluster*[ Title/Abstract] OR modeling[Title/Abstract] OR mapping[Title/Abstract])

AND

(chronic diseases[MeSH Terms] OR chronic illness[MeSH Terms] OR integrated chronic care[Title/Abstract])

AND

(delivery of health care, integrated[MeSH Terms] OR clinical practice pattern[MeSH Terms] OR clinical pathway[MeSH Terms] OR critical pathway[MeSH Terms] OR clinical course[Title/Abstract] OR integrated care[Title/Abstract] OR care map[Title/Abstract] OR care pathway[Title/Abstract] OR care plan[Title/Abstract] OR treatment plan[Title/Abstract] OR disease management[MeSH Terms] OR disease management[Title/Abstract] OR care management, patient[MeSH Terms] OR patient journey[Title/Abstract] OR patient flow[Title/Abstract] OR clinical redesign[Title/Abstract] OR integrated care[Title/Abstract]))

**SCOPUS**

(TITLE-ABS-KEY(“data-driven”) OR TITLE-ABS-KEY("health information") OR TITLE-ABS-KEY("data analys*") OR INDEXTERMS("computer graphics") OR TITLE-ABS-KEY(“visuali*ation”) OR INDEXTERMS("machine learning") OR INDEXTERMS("data mining") OR INDEXTERMS("clinical decision support systems") OR INDEXTERMS("medical informatics application") OR INDEXTERMS("algorithm") OR TITLE-ABS-KEY("supervised learning") OR TITLE-ABS-KEY("unsupervised learning") OR INDEXTERMS("cluster analysis") OR INDEXTERMS(“practice-based”) OR INDEXTERMS("electronic health record") OR TITLE-ABS-KEY("clinical decision support systems") OR TITLE-ABS-KEY("process mining") OR TITLE-ABS-KEY("data mining") OR TITLE-ABS-KEY("machine learning") OR TITLE-ABS-KEY("medical informatics application") OR TITLE-ABS-KEY(cluster*) OR TITLE-ABS-KEY("modelling") OR TITLE-ABS-KEY("mapping"))

AND (INDEXTERMS("chronic diseases") OR INDEXTERMS("chronic illness") OR TITLE-ABS-KEY("integrated chronic care"))

AND (INDEXTERMS("integrated delivery of health care") OR TITLE-ABS-KEY("clinical practice pattern") OR INDEXTERMS("clinical pathway") OR INDEXTERMS("critical pathway") OR TITLE-ABS-KEY("clinical course") OR TITLE-ABS-KEY("integrated care") OR TITLE-ABS-KEY("care map") OR TITLE-ABS-KEY("care pathway") OR TITLE-ABS-KEY("care plan") OR TITLE-ABS-KEY("treatment plan") OR INDEXTERMS ("disease management") OR TITLE-ABS-KEY("disease management") OR INDEXTERMS("patient care management") OR TITLE-ABS-KEY("patient journey") OR TITLE-ABS-KEY("patient flow") OR TITLE-ABS-KEY("clinical redesign") OR TITLE-ABS-KEY("integrated care"))

**IEEE**

(((((((((((((((((((((((((((((("Index Terms":electronic health records) OR "Index Terms":data mining) OR "Index Terms":machine learning) OR "Index Terms":clinical decision support systems) OR "Index Terms":cluster analysis) OR "Index Terms":medical informatics) OR "Index Terms":computer graphics) OR "Index Terms":algorithm) OR "IEEE Terms":medical information systems) OR "IEEE Terms":electronic medical records) OR "Author Keywords":healthcare practices) OR "Publication Title":data-driven) OR "Abstract":data-driven) OR "Publication Title":machine learning) OR "Abstract":machine learning) OR "Publication Title":cluster analys*) OR "Abstract":cluster analys*) OR "Publication Title":data mining) OR "Abstract":data mining) OR "Author Keywords":electronic health record) OR "IEEE Terms":Guidelines) OR "IEEE Terms":Data mining) OR "IEEE Terms":Algorithm design and analysis) OR data mining) OR data-driven) OR electronic health record) OR algorithm) OR visualization) OR clustering) OR algorithm)

AND ((((((((((("Index Terms":clinical pathway) OR "Publication Title":clinical pathway) OR "Abstract":clinical pathway) OR"Author Keywords ":clinical pathway) OR "Author Keywords":healthcare practices) OR"Author Keywords ":Pathway) OR clinical path*) OR care pattern) OR care plan) OR care map) OR critical path*)

AND ((((("Index Terms":chronic disease) OR "Publication Title":chronic disease) OR "Abstract":chronic disease) OR "IEEE Terms":Diseases) OR chronic*)

**EMBASE OR CINAHL**

((Electronic health record or data mining or machine learning or clinical decision support systems or analysis, cluster or medical informatics application).sh. or (Data-driven or visualisation or computer graphics or process mining or data mining or visualization or supervised learning or unsupervised learning or practice based or modelling or mapping or cluster* or data analys*).ti. or (Data-driven or visualisation or computer graphics or process mining or data mining or visualization or supervised learning or unsupervised learning or practice based or modelling or mapping or cluster* or data analys*).ab.)

and

((Clinical pathways or delivery of health care, integrated or clinical practice pattern or disease management or care management, patient).sh. or (Clinical course or integrated care or care map or care pathway or care plan or treatment plan or patient journey or patient flow or clinical redesign or integrated care).ti. or (Clinical course or integrated care or care map or care pathway or care plan or treatment plan or patient journey or patient flow or clinical redesign or integrated care).ab.)

((Electronic health record or data mining or machine learning or clinical decision support systems or analysis, cluster or medical informatics application).sh. or (Data-driven or visualisation or computer graphics or process mining or data mining or visualization or supervised learning or unsupervised learning or practice based or modelling or mapping or cluster* or data analys*).ti. or (Data-driven or visualisation or computer graphics or process mining or data mining or visualization or supervised learning or unsupervised learning or practice based or modelling or mapping or cluster* or data analys*).ab.)

and

((Clinical pathways or delivery of health care, integrated or clinical practice pattern or disease management or care management, patient).sh. or (Clinical course or integrated care or care map or care pathway or care plan or treatment plan or patient journey or patient flow or clinical redesign or integrated care).ti. or (Clinical course or integrated care or care map or care pathway or care plan or treatment plan or patient journey or patient flow or clinical redesign or integrated care).ab.)
