# Supplementary material for: Toward an Interdisciplinary Approach to Constructing Care Delivery Pathways From Electronic Health Care Databases to Support Integrated Care in Chronic Conditions: Systematic Review of Quantification and Visualization Methods
Source: J Med Internet Res. 2023 Dec 14;25:e49996. doi: 10.2196/49996 (PMC10755664; doi:10.2196/49996)
Supplement: Multimedia Appendix 3 [file jmir_v25i1e49996_app3.docx]

## Supplementary material – Critical appraisal – application of the QATSDD

| QATSDD | Warren et al 1999 | Pansaraza et al 2004 | Mabotuwana et al 2010 | Husain et al 2012 | Hsu et al 2012 | Sun et al 2013 | Bettencourt-Silva et al 2015 | Zhang and Padman 2015 | Zhang and Padman 2016 | Zhang and Padman 2017 | Litchfield et al 2017 | Guo et al2018 | Umer et al 2019 | Richter et al 2021 |
| --- | --- | --- | --- | --- | --- | --- | --- | --- | --- | --- | --- | --- | --- | --- |
| Explicit theoretical framework | NA | NA | NA | NA | NA | NA | NA | NA | NA | NA | NA | NA | NA | NA |
| Statement of aims/objectives in main body of report | 3 | 3 | 3 | 3 | 3 | 3 | 3 | 1 | 3 | 3 | 3 | 3 | 3 | 3 |
| Clear description of research setting | 3 | 3 | 3 | 3 | 3 | 3 | 3 | 3 | 3 | 3 | 3 | NA | 3 | 2 |
| Evidence of sample size considered in terms of analysis | NA | NA | NA | NA | NA | NA | NA | NA | NA | NA | NA | NA | NA | 1 |
| Representative sample of target group of a reasonable size | NA | NA | NA | NA | NA | NA | NA | NA | NA | NA | NA | NA | NA | 1 |
| Description of procedure for data collection | NA | NA | NA | NA | NA | NA | NA | NA | NA | NA | NA | NA | NA | 3 |
| Rationale for choice of data collection tool(s) | NA | NA | NA | NA | NA | NA | NA | NA | NA | NA | NA | NA | NA | 0 |
| Detailed recruitment data | NA | NA | NA | NA | NA | NA | NA | NA | NA | NA | NA | NA | NA | 1 |
| Statistical assessment of reliability and validity of measurement tool(s) (Quantitative only) | NA | NA | NA | NA | NA | NA | NA | NA | NA | NA | NA | NA | NA | NA |
| Fit between stated research question and method of data collection (Quantitative) | NA | NA | NA | NA | NA | NA | NA | NA | NA | NA | NA | NA | NA | 2 |
| Fit between stated research question and format and content of data collection tool e.g. interview schedule (Qualitative) | NA | NA | NA | NA | NA | NA | NA | NA | NA | NA | NA | NA | NA | 0 |
| Fit between research question and method of analysis | NA | NA | NA | 3 | NA | NA | NA | NA | NA | NA | NA | NA | NA | 1 |
| Good justification for analytical method selected | NA | NA | NA | 3 | NA | NA | NA | NA | NA | NA | NA | 3 | NA | 1 |
| Assessment of reliability of analytical process (Qualitative only) | NA | NA | NA | NA | NA | NA | NA | NA | NA | NA | NA | 0 | NA | 0 |
| Evidence of user involvement in design | 3 | 0 | 1 | 0 | 1 | 0 | 3 | 0 | 0 | 0 | 3 | 1 | 3 | 3 |
| Strengths and limitations critically discussed | 0 | 1 | 3 | 0 | 0 | 2 | 3 | 0 | 3 | 2 | 3 | 2 | 2 | 3 |
| Score | 0.75 | 0.58 | 0.83 | 0.66 | 0.58 | 0.66 | 1 | 0.33 | 0.75 | 0.66 | 1 | 0.6 | 0.58 | 0.47 |
