# Supplementary material for: Toward an Interdisciplinary Approach to Constructing Care Delivery Pathways From Electronic Health Care Databases to Support Integrated Care in Chronic Conditions: Systematic Review of Quantification and Visualization Methods
Source: J Med Internet Res. 2023 Dec 14;25:e49996. doi: 10.2196/49996 (PMC10755664; doi:10.2196/49996)
Supplement: Multimedia Appendix 4 [file jmir_v25i1e49996_app4.docx]

Multimedia Appendix 3- Clinical aspects of the CDP quantification and visualization methods selected

| Record | Clinical aim | Clinical information used | Relevance criteria | Evaluation criteria^a^ |
| --- | --- | --- | --- | --- |
| Warren et al 1999 | To provide guideline-based elements to help clinicians formulate care plans for patients with complex and chronic illness | Summary display: patient identification data, diagnoses, allergies, and key observations. The key observations vary from project to project. For example, in the cardiac project these are the cardiac risk factors of blood pressure, cholesterol, weight, smoking status and diabetes status.  Services display: Patient's goals, all services available, individualized schedule of services, reminders of cost. | Clinical guidelines: care mentors (specialists, GPs, nurses, and other health professionals) selected 5 key observations to emphasize the most critical information (per project). | Formulation of care plans by care coordinators to fictionalized cases using the system or paper.  Quality of the care plan to be assessed by specialists and scored considering key health risks.  Use assessed by automatic logging of GPs use of system features. User satisfaction assessed using a validated questionnaire. |
| Panzarasa et al 2004 | To help coordinate care in diabetes by enabling primary care actors to communicate between them and with clinical service providers, and handle the overall communication process underlying chronic disease management | Medical history, physical examination, laboratory evaluation (lipid assessment, foot and eye examination, microalbuminuria, others), management plan (including insulin treatment, self-management program including blood glucose monitoring, control visits with GPs, diabetologists, nurses, dieticians, psychologists, and pharmacists). | Example on management of diabetes, relevance is decided following guidelines by the American Diabetes Association, translated into a process model using Workflow Management Coalition (WfMC) standards | - |
| Mabotuwana et al 2010 | To help clinicians detect patients with suboptimal management by enabling them to perform quality auditing using disease-specific criteria, to support assessment of quality improvement programs or to act as a tool to support immediate follow-up | The extracted dataset consisted of demographics (age in years, ethnicity, and gender), prescribing (but not dispensing) details, classifications (diagnosis codes based on Read Clinical Codes, and some procedure codes), relevant laboratory test results, and blood pressure measurements. | Collaboration with two New Zealand general practices; series of iterative discussions with an expert panel from one of the clinics (practice manager, three GPs and two nurses) | - |
| Husain et al 2012 | To improve diagnostic and referral pathways by 1) Retrospective and prospective tracking of patient pathways, 2) Validation of patient-reported recall data, 3) Objective measure of the cost of illness, 4) Healthcare pathways and referral history, and 5) Profiling of patients | Linking with GP data systems provides healthcare utilization history including previous diagnoses, referrals, presenting symptoms, investigation results and previous medications, associated conditions and use of co-medications; linkage with IP data provides hospital visits, surgery and hospital treatment; linkage with mortality datasets ensure dataset remains relevant; linkage with A&E give information on emergency visits; patient completed questionnaires and trial data. | Not detailed | Possible assessment of the impact of  specific healthcare interventions on subsequent healthcare utilization (e.g., A&E visits or hospital admission) thanks to the longitudinal routine data. |
| Hsu et al 2012 | To help decision making by selecting relevant information in the patient's EHR | Radiology reports (with images), pathology, oncology notes, consultation letters, surgical notes, admission/discharge summaries, and laboratory results. | All information reported for a given disease, iterative development process with physicians. | Performance of physicians is compared when using AdaptEHR versus existing means (e.g., manually generated slide presentation) while reviewing a case at a tumor board conference. Evaluation metrics will include time spent to prepare the case, ability to answer questions posed by board members (e.g., precision/recall of information retrieved from the patient record), and overall satisfaction with the interface. |
| Sun et al 2013 | To enable intelligent care delivery and the detection of patient-specific risk factors within a care coordination system designed to empower a collaborative workforce to improve the health and care for patients with hypertension, heart failure, or diabetes. | Demographics, diagnoses, medications, laboratory results, BP measurements; traditional data from electronic health records supplemented with physician judgement of hypertension control status. | The model uses all available clinical information and physician inputs on BP control, a feature selection strategy is applied to identify characteristics relevant to the transition prediction algorithm. | - |
| Bettencourt-Silva et al 2015 | To select high quality data for clinical studies and decision making; enable (re)design, management, and optimization of pathways | Patient-centric data conveyed in in workflow logs produced by multiple hospital information systems or EHR, and local cancer registry; a holistic representation of the patients, including their demographics, comorbidities, test results, or other information, and is limited by the availability of electronic information in the hospital information systems. In the case study on prostate cancer, the operational data store contains information from the following systems: administration, cancer waiting times, histopathology, radiology, biochemistry, operating theater, orthopedics, oncology, and radiotherapy. | Selection of specific data elements from the operational data store to form a pathway performed later in consultation with the domain experts (urology consultant, prostate cancer geneticists, a consultant oncologist, a histopathologist, and a chemical pathologist). | - |
| Zhang and Padman 2015 | To provide visibility into ways to improve patients’ experience during treatments, examine treatment approaches and quality of care, and foster innovative care models in healthcare practices based on treatment data | Multidimensional EHR data about patients’ visits to the clinic, including the visit purpose, procedures, medications, and diagnoses, transformed into a sequence of visits represented by a one-dimensional Markov chain; combinations of the medication, visit purpose, procedure, and diagnosis nodes form super nodes representing the visit content | Developed in collaboration with a specialist/clinic; no further details | - |
| Zhang and Padman 2016 | To achieve accurate predictions of anticipated future events and costs following different clinical and cost pathways for improved shared decision making | Previous studies: a) encounters; b) diagnoses as International Classification of Diseases, Ninth Revision, Clinical Modification (ICD-9-CM) codes; c) procedures as Current Procedural Terminology (CPT) codes; and d) prescriptions of medications summarized by drug classes. This study combines statistical machine learning and data visualization by specifically incorporating costs associated with medications. | Information from previous studies by the same authors developed in collaboration with a specialist/clinic and addition of costs | - |
| Zhang and Padman 2017 | To support shared decision making in the context of multiple chronic conditions | Encounter types, diagnoses, and interventions, recorded at different points in time | Developed in collaboration with a specialist/clinic; no further detail | Evaluation of the platform with actual users in a formal experimental setting, such as through think-aloud protocols and focus groups, is considered by the authors to be important future work. To accommodate  this task, they will work on adding human computer  interaction capabilities, such as recording the number of  times a node is clicked by a user in order, to understand what features users are most interested in. |
| Litchfield et al 2017 | (Protocol) To understand and optimize organizational performance in the management of two chronic conditions (hypertension and type 2 diabetes) | Coded information on patient contact with the service including consultations, diagnoses, prescriptions and laboratory tests, events (prescriptions, referrals, appointments, etc.) recorded with a date. | Existing algorithms as a starting point for process mining, iterative interaction with experts to validate results; focus groups with clinical and non-clinical staff and patients for process mapping. | Automatization of care patterns by comparing process models generated by process mining techniques in primary care settings to process maps generated using traditional process mapping methods. |
| Guo et al 2019 | To improve data interpretation by providing visualization of event sequences | Application of the method to a public critical care dataset (MIMIC), containing de-identified electronic health records with timestamped events organized into seven categories (admission and discharge, death, ICU admission and discharge, prescriptions, infusions, laboratory tests, and microbiological tests) | Authors' experience in the field, a review of the existing features and limitations of existing tools, and interviews with domain expert users | - |
| Umer et al 2019 | To help improve clinicians' confidence when deciding whether a decision suggested by a decision support system is appropriate by providing visualizations and predicting probable patient outcomes | Six core data entities: patient, injury, situation, condition, measurement and treatment related to each other with one-to-many relationship; and many specialized sub-types/entities, like CT, MRI and Glasgow Coma Scale measurements (measurement entities) or pharmacology and surgery (treatment entities) | Specifically designed with clinicians to address data analytics challenges in clinical practice in an iterative process of design and development | Comparison of predicted outcomes (unfavorable/favorable outcome) by clinicians with/without the DSS |
| Richter et al 2021 | To enable a more efficient and effective use of available data by the orchestration of available information for a patient in the continuum of care—consisting of hospitals, outpatient departments, practices, non-physician health-service providers, home monitoring—into a comprehensive view | Data from multiple sources such as hospitals, medical practices, insurance companies, and research facilities - no further detail. | User requirements were identified in focus groups comprising various stakeholders (e.g., patients,  physicians, study nurse, health care insurance) and patient interviews. | Functional capacity (Functional Ability Questionnaire (FFbH)), and its values were derived to Health Assessment Questionnaire (HAQ). Self-reported disease activity (RA Disease Activity Index (RADAI)). Disease Activity Score (DAS) 28 CRP, and medication were recorded at baseline and follow-up visits (after 3 and 6 months). |

^a^Evaluation criteria were proposed for future work and not included in the research reported in the selected studies (except for Richter et al, 2021).
